# Research advances on signaling pathways regulating the polarization of tumor-associated macrophages in lung cancer microenvironment

**DOI:** 10.3389/fimmu.2024.1452078

**Published:** 2024-07-31

**Authors:** Wenqiang Li, Quan Yuan, Mei Li, Xiaoyu He, Chen Shen, Yurui Luo, Yunze Tai, Yi Li, Zhiping Deng, Yao Luo

**Affiliations:** ^1^ Department of Respiratory and Critical Care Medicine, Zigong First People’s Hospital, Zigong, Sichuan, China; ^2^ West China Hospital, Sichuan University, Chengdu, Sichuan, China; ^3^ Department of Clinical Medicine, North Sichuan Medical College, Nanchong, Sichuan, China

**Keywords:** LC, TME, TAMs, polarization, NDDSs

## Abstract

Lung cancer (LC) is one of the most common cancer worldwide. Tumor-associated macrophages (TAMs) are important component of the tumor microenvironment (TME) and are closely related to the stages of tumor occurrence, development, and metastasis. Macrophages are plastic and can differentiate into different phenotypes and functions under the influence of different signaling pathways in TME. The classically activated (M1-like) and alternatively activated (M2-like) represent the two polarization states of macrophages. M1 macrophages exhibit anti-tumor functions, while M2 macrophages are considered to support tumor cell survival and metastasis. Macrophage polarization involves complex signaling pathways, and blocking or regulating these signaling pathways to enhance macrophages’ anti-tumor effects has become a research hotspot in recent years. At the same time, there have been new discoveries regarding the modulation of TAMs towards an anti-tumor phenotype by synthetic and natural drug components. Nanotechnology can better achieve combination therapy and targeted delivery of drugs, maximizing the efficacy of the drugs while minimizing side effects. Up to now, nanomedicines targeting the delivery of various active substances for reprogramming TAMs have made significant progress. In this review, we primarily provided a comprehensive overview of the signaling crosstalk between TAMs and various cells in the LC microenvironment. Additionally, the latest advancements in novel drugs and nano-based drug delivery systems (NDDSs) that target macrophages were also reviewed. Finally, we discussed the prospects of macrophages as therapeutic targets and the barriers to clinical translation.

## 1 Introduction

Lung cancer (LC) is the most common malignant tumor with the highest incidence and mortality rates globally, seriously threatening human health (1). Tumors involve an ongoing struggle, adaptation, and evolution among tumor cells, surrounding normal cells, and various immune cells. Tumor microenvironment (TME) plays an important role in various stages of tumor (2). Additionally, the composition of the TME is rich and heterogeneous, including various cellular components and signaling molecules (3). Among patients with LC, these components can also serve as important biomarkers for auxiliary diagnosis, treatment efficacy evaluation, and prognosis assessment (4–7).

Tumor-associated macrophages (TAMs) mainly originate from peripheral blood monocytes and tissue-resident macrophages (TRMs) (8, 9). During embryonic development, precursor cells from the yolk sac or fetal liver migrate into various organ tissues, where they differentiate into tissue-resident macrophages. Resident macrophages in normal lung tissue include interstitial macrophages (IMs) and alveolar macrophages (AMs) (10). In adulthood, besides tissue-resident macrophages that persist long-term and self-renew, another major source of macrophages in tissues is peripheral blood monocytes (11, 12). In a mouse model of LC, both embryonically-derived TAMs and monocyte-derived TAMs cannot be strictly defined as either M1-like or M2-like macrophages, but rather appear to be a mixture of M1 and M2 phenotypes (13). Monocytes are recruited from the peripheral circulation to the TME through colony-stimulating factor-1 (CSF-1) secreted by tumor cells, and subsequently differentiate into macrophages (14). TAMs are important cellular components of the TME, exerting multiple supportive and inhibitory roles in LC growth and metastasis (15–17). TAMs possess at least seven functions, including promoting tumor growth, metastasis, therapy resistance, stemness maintenance, immune regulation, inflammation, and angiogenesis, which collectively influence tumor progression and treatment outcomes (18). Currently, two functionally distinct main phenotypes of macrophages have received extensive attention, namely the inflammatory or classically activated M1-like TAMs and the anti-inflammatory or alternatively activated M2-like TAMs. On the surface of M1 macrophages, there are expressed markers such as TLR2, TLR4, CD80, CD86, iNOS, and MHC-II, which are characteristic markers of M1 macrophages. In contrast, M2 macrophages express proteins including the mannose receptor, CD206, CD163, CD209, CD204, CD115, CD301, FIZZ1, and Ym1/2 on their surface (19, 20). With the advancement of biological detection technologies, more subtypes of TAMs have been defined. For instance, slight phenotypic variations have been observed within the M2 phenotype, leading to the classification of distinct M2 subgroups known as M2a, M2b, M2c, and M2d, based on the stimuli used for activation (21). However, this classification fails to explain the phenotypic range observed in macrophages under different homeostatic and pathological conditions. Additionally, genes associated with M1-like and M2-like TAM spectra are expressed concurrently in nearly all types of TAMs subgroups. Recently, research employing single-cell analysis identified five distinct TAM subgroups in various cancers: HES1 TAMs, C1Qhi TAMs, TREM2 TAMs, IL4I1 TAMs, and proliferative TAMs (22). TAMs interact with cancer cells, T cells, endothelial cells, B cells, fibroblasts, and NK cells through complex signaling pathways (23–26). In response to signaling crosstalk between TAMs and other cells, an immunosuppressive TME is formed, which promotes cancer cell proliferation, angiogenesis, lymphangiogenesis, and distant metastasis (27–29). Previous studies have shown that M2-like TAMs play three distinct roles in promoting tumor development and metastasis: (1) M2-like TAMs promote tumor cell entry into the vascular system and facilitate tumor spread through paracrine signaling loops (30); (2) M2-like TAMs can promote the formation of an immunosuppressive tumor microenvironment by secreting immunosuppressive factors including TGF-β, IL-10, arginase-1 (Arg-1), and NO, thereby facilitating tumor growth (31); (3) M2-like TAMs can enhance tumor angiogenesis, promoting tumor growth and post-therapeutic repair (32). In the progression of tumors, the immunosuppressive TME can further attract macrophages to accumulate, forming a vicious cycle (33, 34).

With the advancement of multi-omics technologies, various biomolecules have been identified as key signals mediating TAM polarization (34). Epigenetics has introduced novel concepts to comprehend genetics, garnering significant and profound research focus, particularly concerning tumor development. In the past, it was thought that new genetic phenotypes arose from alterations (or mutations) in DNA sequences inherited by the next generation. However, it is now known that epigenetic modifications can transmit new phenotypic traits to offspring without directly modifying the DNA sequence itself. In recent years, reprogramming macrophage plasticity and function through epigenetic mechanisms has been recognized as a key regulatory node upstream of transcriptomics, proteomics, and metabolic processes (2). At the same time, numerous epigenetic signaling pathways have been found to play crucial roles in regulating the TME in LC (35, 36). Drugs targeting macrophage have been shown to effectively inhibit cancer cells, including small molecule inhibitors and monoclonal antibodies (37–39). Additionally, an increasing number of natural drug compounds have been discovered to combat tumors by modulating the immune microenvironment (40–42). Nanomedicine is an emerging product of the combination of nanotechnology and medicine, used for the diagnosis, treatment, and prevention of various diseases including tumors and immune disorders. Nano-drug delivery systems (NDDSs) have the advantages of selectivity and controllability, and its effectiveness in enhancing anti-tumor responses by targeting macrophages has been widely demonstrated *in vitro* (43–47).

**Figure 1:**
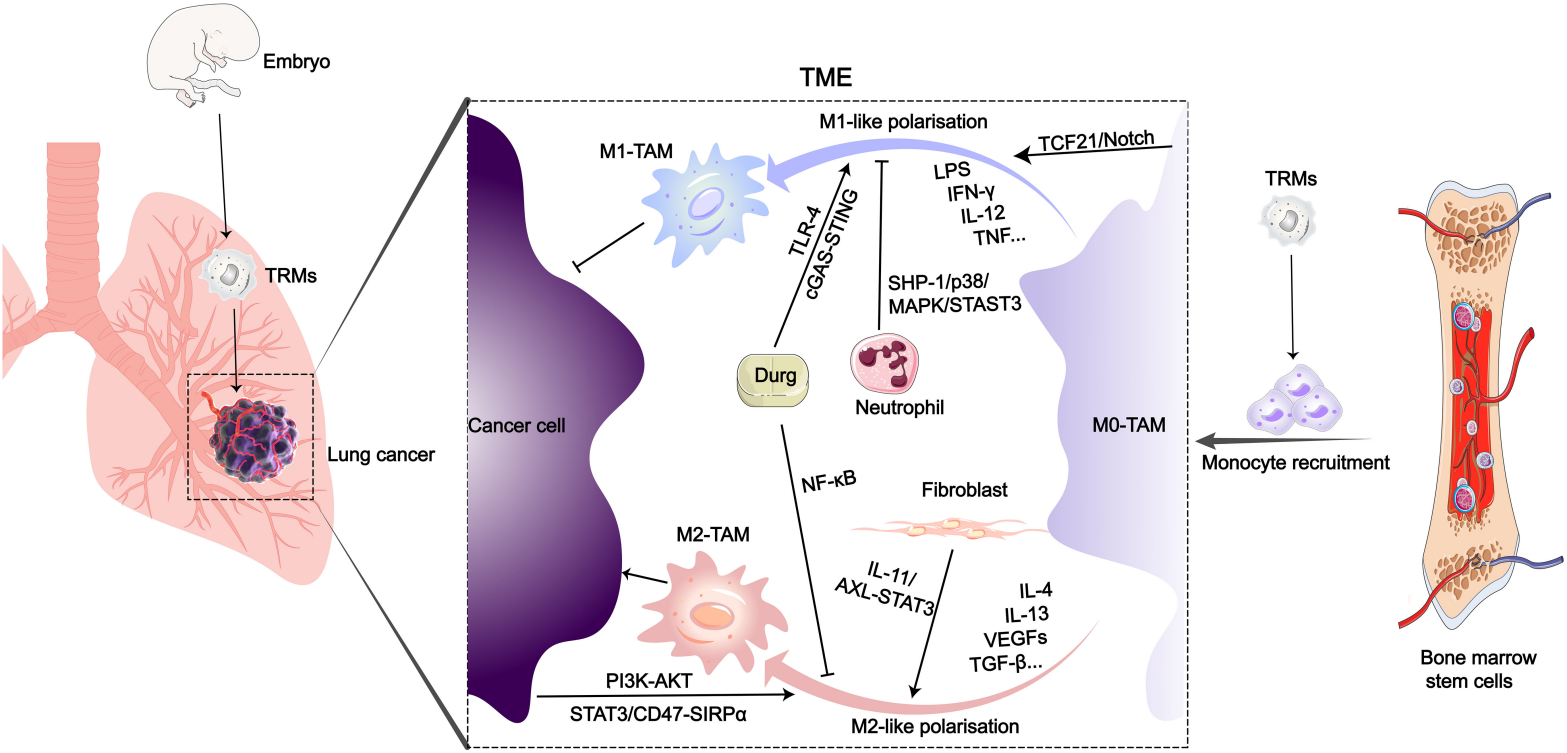
Origin and polarization-related signaling pathways of TAMs in the LC microenvironment. After entering the TME of LC, various biological or drug molecules promote or inhibit monocyte polarization into M1 or M2-like subtypes.

In this review, we primarily summarized the signaling pathways regulating TAM phenotype switching in LC. Based on TAM-related signaling targets, we reviewed the latest advances in NDDSs and novel drugs (**Figure 1**). At the same time, the future prospect of macrophages as therapeutic objects and the existing obstacles were discussed.

## 2 Macrophage phenotypes in LC

**Figure 2:**
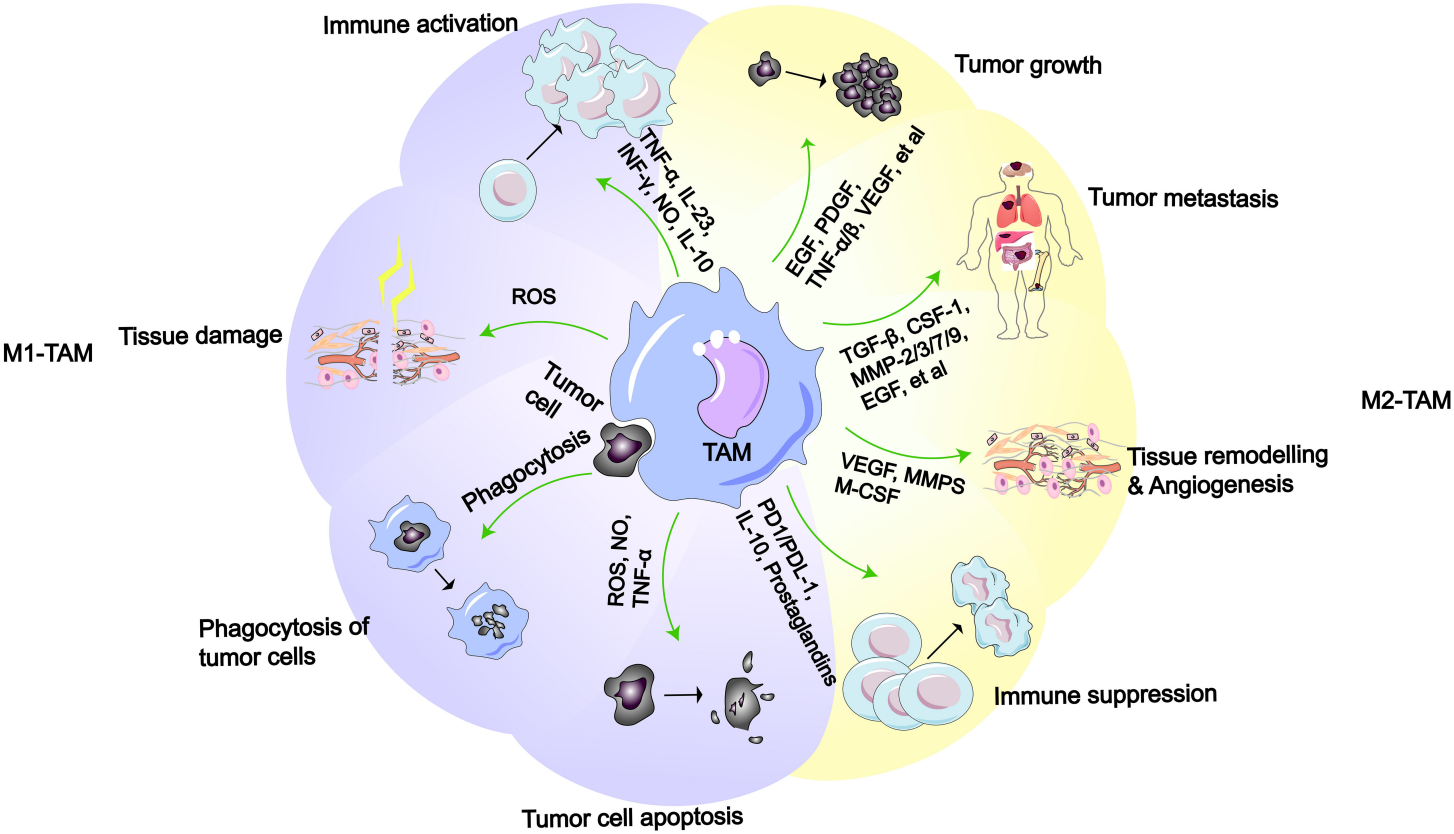
M1-like or M2-like macrophages and their functions (2). M1-like TAMs possess functions such as immune activation, tissue damage, phagocytosis of tumor cell, and promotion of tumor cell apoptosis. M2-like TAMs have functions including promoting tumor growth, metastasis, tissue remodeling/angiogenesis, and immune suppression.

In the TME, TAMs are a principal cellular component, comprising more than half of the total immune cells in the tumor stroma and playing a significant role in tumor progression (48–50). According to the latest research, after macrophages senescence in the lungs, they not only persist, but also become pro-tumorigenic, suppressing the anti-cancer activity of cytotoxic T lymphocytes, ultimately promoting cancer development (51). Furthermore, researchers have found a close association between the senescence-associated secretory phenotype (SASP) secreted by senescent macrophages and *KRAS*-mutant NSCLC (52). In response to the influence of cytokines in the TME, macrophages differentiate into different types, mainly divided into M1-like macrophages and M2-like macrophages (26, 53). In early LC, the same TAMs can concurrently exhibit two distinct gene expression profiles, demonstrating that the expression of M1-like and M2-like gene characteristics at this stage is not mutually exclusive (54). And macrophages aggregate around cancer cells within the tissue, promoting cancer cell invasion (55). Simultaneously, macrophages induce an increase in regulatory T cells, aiding cancer cells in immune evasion (56). In the TME, the process by which macrophages differentiate into M1-like TAMs or M2-like TAMs is referred to as polarization (57). M1-like macrophages are typically regarded as tumoricidal macrophages, primarily involved in anti-tumor responses and immune promotion. Conversely, M2-like macrophages exhibit immunosuppressive properties, promoting tissue repair and tumor growth (**Figure 2**). According to reports, the polarization of TAMs in the TME is associated with different outcomes in LC patients, with a higher proportion of M2-like TAMs being correlated with a poorer survival time (6, 15, 58, 59). Additionally, TAM polarization leads to the widespread presence of M2 macrophages in the TME, which can induce LC cells to develop drug resistance (60, 61).

With the development of technologies such as metabolomics and single-cell sequencing, more molecular signaling pathways regulating TAM polarization have been discovered, providing additional insights into the evolution of tumor cells and drug development.

## 3 Signaling between LC cells and macrophages

LC cells and TAMs exhibit complex signal crosstalk (62). LC is prone to genetic mutations, and many signaling pathways related to macrophage polarization have been discovered among the common types of gene mutations (63). Cellular senescence is a process characterized by cell cycle arrest accompanied by changes in gene expression, but with normal cell viability and metabolism, associated with cancer and aging (64, 65). M2-like TAMs are associated with high expression of IL-10, IL-1β, VEGF, and matrix metalloproteinases (MMPs) *in vivo*. What’s more, they also express abundant scavenger receptors, contributing to debris clearance, promotion of angiogenesis, tissue remodeling and repair, as well as facilitating tumor initiation and progression (66). Among these, *VEGF* exerts the strongest and most specific effects, not only promoting proliferation and neovascularization of endothelial cells but also increasing vascular permeability, facilitating the extravasation of tumor cells (67). Furthermore, TAMs can promote the formation of collagen fibers, facilitating rapid migration of LC cells along these fibers towards blood vessels, thereby enhancing the metastatic efficiency of LC cells (68). M2-like macrophages enhance cancer cell drug resistance by producing and releasing mediators that modulate the PI3K/Akt, JAK/STAT, MAPK, Notch, Hippo, and other related pathways (69). Extracellular vesicles derived from M2-TAMs promote aerobic glycolysis in LC cells *via* the miR-3679–5p/NEDD4L/c-Myc signaling pathway, thereby inhibiting tumor cell apoptosis and inducing cisplatin resistance (70). The TF-mTORC2 axis plays a role in negative regulation of macrophage phagocytic capacity, contributing to *KRAS-*driven lung adenocarcinoma (LUAD) resistance (71). M2-like TAMs promote osimertinib resistance in NSCLC through extracellular vesicles that regulate the miR-6386–5p/MAPK8IP3 axis (72).

*KRAS*-driven LC is characterized by senescent endothelial cells and macrophages (51, 52). Meanwhile, due to activation of the AKT-mTOR signaling pathway, ubiquitin-specific protease-12 (USP12) is typically downregulated, promoting both cancer cells growth and macrophages recruitment (73). In addition, in *KRAS-*mutant LC, activation of the PI3K/STAT3 signaling pathway can lead to increased CD47 expression, thereby reducing macrophage phagocytic capability (74). In *KRAS*-mutated tumor cells, the mechanistic study indicates that endogenous anti-silencing function protein 1 homolog A (Asf1a) deficiency induces polarization of M1-like macrophages by upregulating GM-CSF expression (75). In *KRAS*-mutant LUAD, LKB1 deficiency leads to increased expression of the MCT4 transporter protein, ultimately promoting lactate production and secretion (76). Fascin actin-bundling protein 1 (FSCN-1) activates the PI3K-AKT and JAK-STAT signaling pathways in LUAD cells, leading to increased lactate production (77). In acidic environments, macrophages become more polarized towards an M2-like phenotype (76–78). Additionally, low levels of lactate promote TAM maturation, which is regulated by the Notch/Hes1/MCT2 signaling pathway (79). In LC with activated *epidermal growth factor receptor* (*EGFR*) mutation, overexpression of immunoglobulin-like transcript (ILT) 4 in tumor cells induces recruitment of TAMs and M2-like polarization (80). Another study confirmed that the activation of the STAT3/CD47-SIRPα signaling axis promotes an increase in M2-like macrophages, ultimately leading to acquired resistance to *EGFR-*TKIs (61). In small cell lung cancer (SCLC), the Slit2/Robo1 signaling pathway inhibits the polarization of M2-like macrophages in the TME by suppressing the TGF-β1 signaling pathway (81).

**Figure 3:**
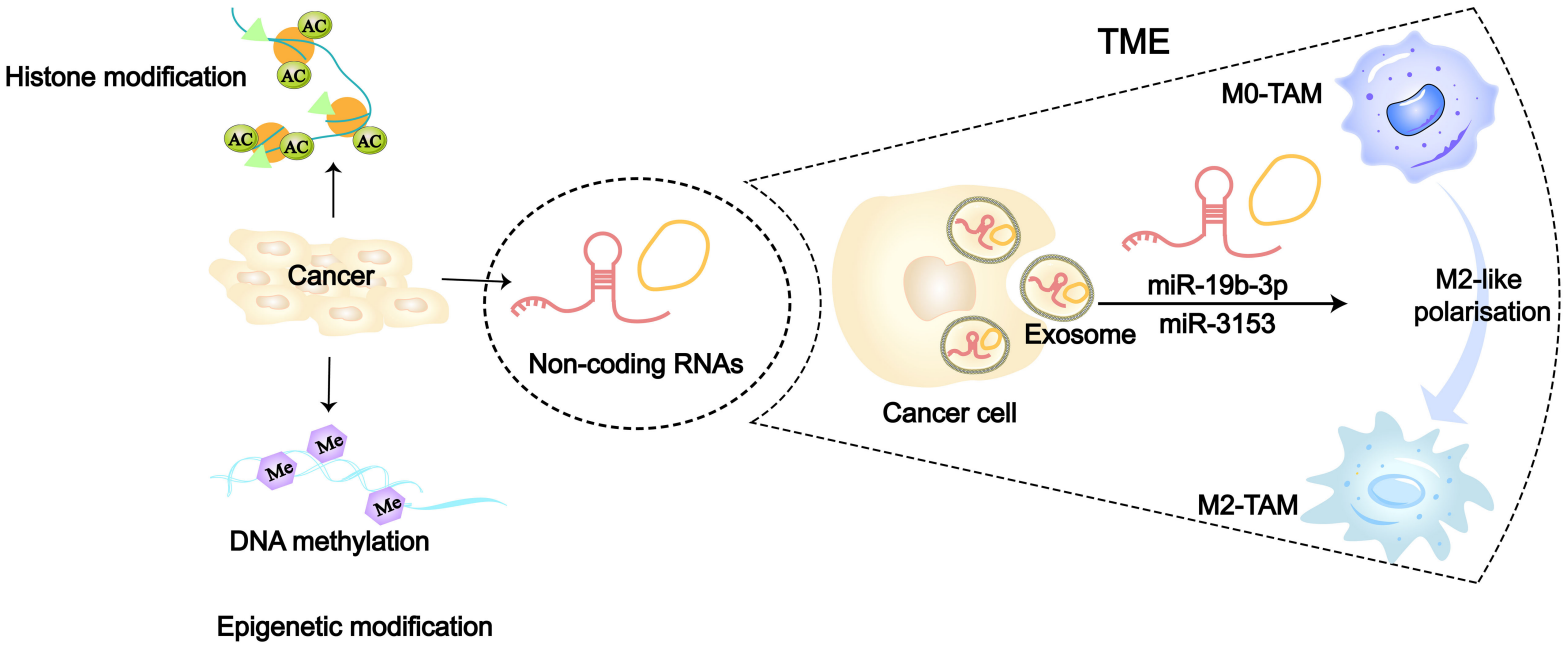
Epigenetic modifications including DNA methylation, histone modifications and non-coding RNA (86). 36126898 Tumor cells can regulate TAMs towards M2-like polarization by transporting ncRNAs (such as miR-315 and miR-19b-3p) via secretion of exosomes.

The traditional view holds that cancer originates from the accumulation of somatic cell mutations. However, in recent years, many studies have found that the occurrence and susceptibility of cancer are also closely related to extensive epigenetic changes in the genome (82–84). Researchers have confirmed for the first time in model organism that transient epigenetic changes are sufficient to induce irreversible cancer transformation, which is associated with the irreversible derepression of certain cancer-driving genes (85). At present, three major epigenetic codes have been extensively studied, including DNA methylation, non-coding RNA (*nc*RNA) and histone modification (**Figure 3**). Many cancer-related gene mutations occur predominantly in non-coding regions. Restricting research focus to the minority of protein-coding genes would severely limit our understanding of cancer initiation and evolution mechanisms. In recent years, increasing evidence has also demonstrated the close association between non-coding RNAs (ncRNAs) and cancer (87). Epigenetic alterations can serve as biomarkers for cancer and even become targets for cancer therapy (88). In LC, the tumor-derived exosomes (TDEs) carrying epigenetic factors also play a significant role in the interaction between cancer cells and macrophages, especially *nc*RNAs (36, 78). *Mi*RNAs in *nc*RNAs have gained more attention due to their relative stability, small size, and high conservation. At the same time, *mi*RNAs are abundantly present and stably expressed in human serum, plasma, and sputum, and their detection in peripheral blood of tumor patients has become a hot topic (89, 90). In NSCLC researches, numerous signaling pathways related to *mi*RNAs have been found to be involved in macrophage polarization. Cancer cells secrete exosomes containing *mi*R-19b-3p, which targets the protein tyrosine phosphatase receptor type D (PTPRD) in TAMs, inhibiting the dephosphorylation of STAT, thereby leading to M2-like polarization (91). Additionally, exosomes secreted by LAUD can activate the c-Jun N-terminal kinase (JNK) signaling pathway through *mi*R-3153, thereby inducing M2 macrophage polarization (92). Exosomes derived from LC promote M2-like macrophage polarization through the *mi*R-124–3p/EZH2 signaling pathway mediated by circPVT1 (93). *In vitro* study of LC, circular RNA (*circ*ATP9A), when bound to HuR, induces M2-like polarization of macrophages by affecting downstream pathways mediated by the PI3K/AKT/mTOR axis (94). Hypoxic LUAD cells activate the STAT3 signaling pathway via miR-1290 to promote M2-like macrophage polarization (95). Morrissey et al. demonstrated in mouse models of LC that TDEs polarize macrophages into an immunosuppressive phenotype (M2-like) through NF-κB signaling (78). Liang et al. found that exosomes (TRIM59) can convert macrophages into M2-like phenotype by regulating ABHD5-mediated proteasomal degradation (96). In poorer prognosis SCLC, cancer cell-derived exosomes induce M2-like macrophages polarization through the NLRP6/NF-κB signaling axis (97).

Furthermore, *in vivo* or *in vitro* experiments, additional signaling pathways involved in regulating macrophages polarization have also been discovered. A study has shown that LC cells release succinate into microenvironment, activating succinate receptor (SUCNR1) signaling, which polarizes macrophages into the M2-like (98). Rab37, dependent on GTPase, regulates macrophage IL-6 secretion, thereby inducing M2-like polarization (99). Human endogenous retrovirus-H long terminal repeat-associating protein 2 (HHLA2) is upregulated in various tumors (100, 101). In NSCLC, HHLA2 can promote M2 polarization by regulating IL-10 (102). NADPH oxidase 4 (NOX 4) promotes NSCLC cell growth by recruiting immunosuppressive macrophages through ROS/PI3K signaling to produce cytokines (103). Estrogen receptor alpha (ERα) can activate the CCL2/CCR2 signaling pathway, promoting macrophage infiltration and M2-like polarization (104). Upregulation of the JAK-STAT and NF-κB signaling pathways can lead to the aggregation of M2-like macrophages (60). The expression of placental growth factor (PIGF) in LC cells can bind to the fms-like tyrosine kinase-1 (Flt-1), promoting the generation of M2-like macrophages (105). Altered expression of solute carrier family 3 member 2 (SLC3A2) changes the levels of various metabolites in the TME, including arachidonic acid. More importantly, in LC, arachidonic acid is responsible for mediating SLC3A2-induced polarization of macrophages to the M2-like (106). Gamma-aminobutyric acid (GABA) originating from LC cells inhibits both the NF-κB and STAT3 pathways, halting the polarization of M1-like phenotype. Simultaneously, it promotes macrophage M2-like polarization by activating the STAT2 pathway (107).

In view of the above, we can design different drugs against tumors or reduce drug resistance by targeting these molecular signaling pathways that influence macrophage polarization. Besides, epigenetic signaling pathways are involved in the polarization of macrophages, particularly *mi*RNAs. It might be a feasible approach to treat LC by selectively regulating the aberrantly expressed miRNAs, which exist in various cancers in differential expression levels. Meanwhile, we can utilize exosomes as biomarkers, vaccines and drug delivery vehicles for LC diagnosis and treatment, but it remains a challenge that identifying, isolating and quantifying exosomes accurately, efficiently and selectively.

## 4 Macrophages in self-polarization

New findings have also been made regarding the signaling molecules that regulate polarization within macrophages. STAT protein family, comprised of transcription factors, is associated with the development, progression, metastasis, survival, and drug resistance of human cancers (108–111). Specifically, STAT3 and STAT6 are significant in cancer biology (108, 112, 113). In the preceding context, many STAT-related signaling pathways involved in macrophage polarization have already been summarized. In TAMs, the upregulation of the epigenetic modulator JMJD6 can be mediated by the STAT3/IL-10 signaling pathway, which can promote the emergence of an immunosuppressive phenotype in TAMs (17). In CD11b cells, the IL-4/STAT6 signaling axis promotes LC progression by increasing M2-like macrophages (114). Furthermore, the AKT/STAT6 signaling pathway can increase the expression of macrophage SLC7A11, promoting TAMs’ M2-like polarization (112). Zhao and colleagues demonstrated that Takeda G protein-coupled receptor 5 (TGR5) promotes the pro-tumoral M2-like polarization in TME by activating the cAMP-STAT3/STAT6 signaling pathway (115). N6-adenosine methylation is one of the most common modifications in RNA. YTHDF2 serves as a primary m6A reader, mediating mechanisms that promote the formation of TAMs (116). N6-methyladenosine (m6A) sequencing results in macrophages show that the absence of METTL3 promotes the activation of NF-kB and STAT3 via the ERK pathway, resulting in the polarization of M2-like macrophages (117). Zhang et al. discovered that GRP78 in macrophages promotes M2-like polarization through the JK/STAT6 pathway, and that the expression of insulin-like growth factor 1 (IGF 1) within macrophages has a similar effect (118). In various malignant tumors and immune cells, the transcription factor β-catenin signaling is often highly expressed (119, 120). In NSCLC, TAM-specific Wnt/β-catenin signaling plays a role in immune evasion by mediating the transformation of M1-like TAMs to M2-like TAMs (121). Research by Sarode et al. also demonstrated that β-catenin-mediated transcriptional activation of FOS-like antigen 2 (FOSL2) drives gene regulation that shifts from M1-like TAMs to M2-like TAM phenotypes. Similarly, the inhibition of the AT-rich interaction domain 5A (ARID5A) has a similar effect on the regulation of macrophage polarization (121). In SCLC, SLIT2 promotes M1 polarization by regulating the Tgf-β1/GSK3/β-catenin signaling pathway in TAMs (81). *EGFR*/Wnt/β-catenin upregulation of CD55/CD59 expression negatively correlates with the infiltration of M1 macrophages (122). In NSCLC, the transcription factor c-Maf is expressed in TAMs, where it controls oxidative phosphorylation and the N-glycan synthesis pathway, thereby reprogramming macrophages to an M2-like phenotype (123).

Ubiquitination and deubiquitination processes participate in various cellular events through immune regulation, signal transduction (124, 125). Among them, USP7 is identified as a highly expressed gene in M2 macrophages, where it inhibits the polarization of M2-like macrophages by activating the p38 MAPK pathway. Simultaneously, targeting USP7 can significantly promote the polarization of TAMs towards M1-like (126). Inactivation of EGLN3 in macrophages inhibits their migration, phagocytosis, and polarization towards the M2-like phenotype (127). Overexpression of lncRNA ADPGK-AS1 in TAMs upregulates the tricarboxylic acid cycle and promotes mitochondrial fission, indicating a shift towards an M2-like phenotype (128). Therefore, silencing ADPGK-AS1 in TAMs may provide a novel therapeutic strategy for LC. Li et al. identified a novel Mincle/Syk/NF-κB signaling pathway in TAMs, the blockade of which promotes an M1-like phenotype (129). TAMs-associated TMSB10 enhances the transformation and proliferation of M2 TAMs via the PI3K/Akt signaling pathway (130). Transcription factor 21 (TCF21) exerts its effects via the Notch signaling pathway, and its overexpression promotes polarization of TAMs towards M1 macrophages (131). JMJD6-induced M2 polarization in TAMs is mediated by STAT3/IL-10 signaling transduction (17). The 78 kilodalton glucose regulated protein (GRP78) reduces macrophage M1 polarization and promotes M2 polarization through JAK/IGF-1 (118). Histone deacetylase 2 (HDAC2) in macrophages regulates the M2-like phenotype through acetylation of histone H3 and transcription factor SP1 (15).

## 5 Signaling crosstalk between macrophages and non-tumor cells in TME

In the microenvironment of LC, there is complex signaling crosstalk between macrophages and non-tumor cells (132, 133). It has been demonstrated that cancer-associated fibroblasts (CAFs) are derived from macrophages of the M2-like, which are known as macrophage-to-mesenchyme transitions (MMTs). Meanwhile, Smad3 has been found to play a crucial role in MMT of LC (134). Wu et al. confirmed that CAFs secrete CXCL12 to mediate M2-polarized macrophages (135). Interleukin-11 (IL-11) secreted by CAF triggers the AXL-STAT3 signaling pathway, which can differentiate TAMs into a phenotype reminiscent of “M2-like” (136). Additionally, in heavy smokers, *mi*R-320a secreted by neutrophils downregulates STAT4 upon entering macrophages, promoting the accumulation of M2-like pro-tumorigenic phenotypes (137). Upregulating the expression of signal regulatory protein-alpha (SIRPα) in neutrophil-like cells regulates the SHP-1/p38/MAPK/STAT3 signaling cascade, reducing M1-like macrophages, while lacking SIRPα decreases the polarization of M2-like macrophages (138). IL-17A/F secreted by Th17 cells promotes LC metastasis by inducing polarization of macrophages towards an M2-like phenotype (139). However, TNFSF15 secreted by vascular endothelial cells inhibits STAT6, which leads to macrophages towards M1-like (140).

## 6 Drugs that modulate macrophage phenotypes

**Figure 4:**
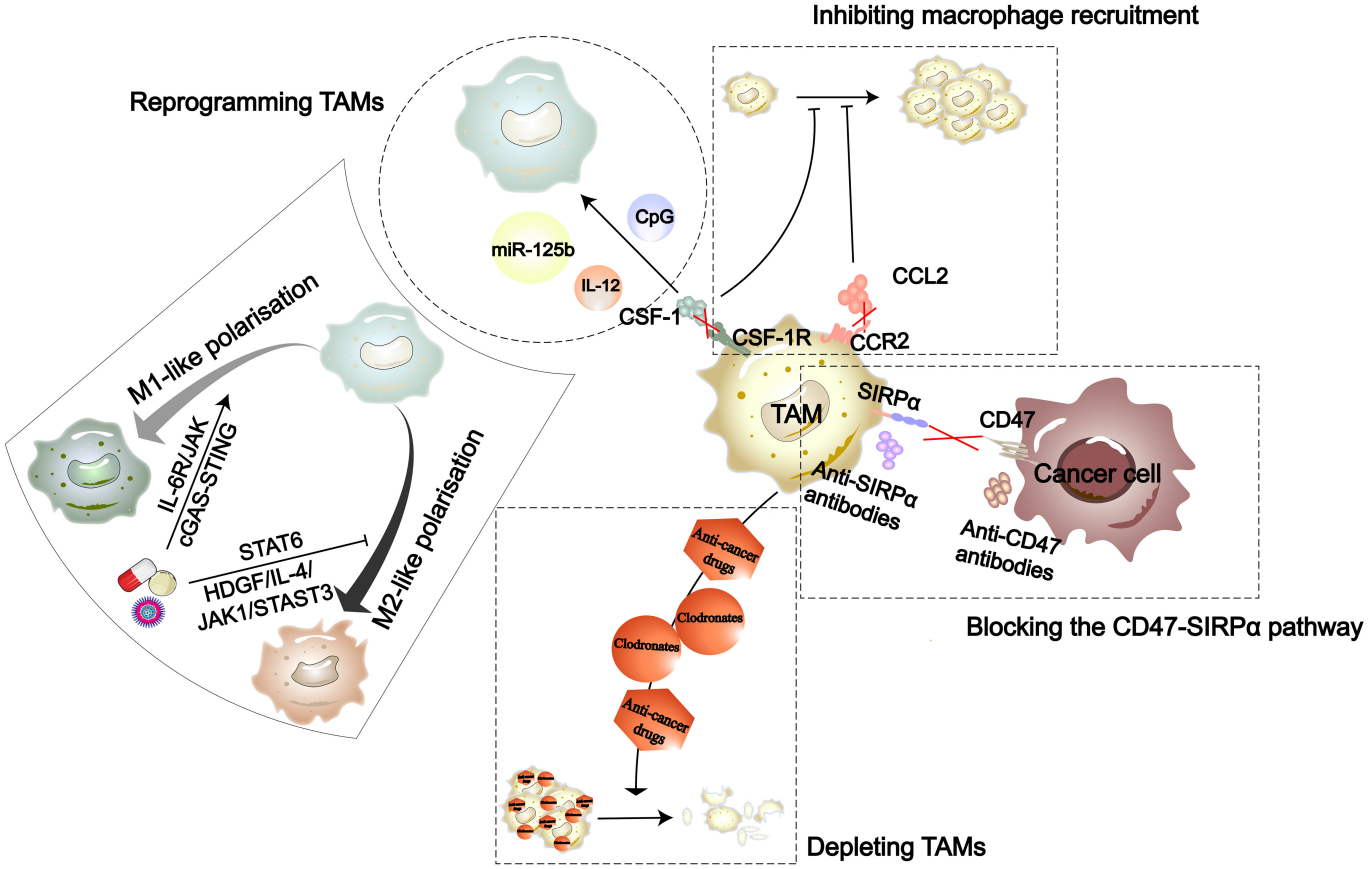
The strategies in TAM-targeted therapy include inhibiting recruitment of macrophages, reprogramming TAMs, depleting TAMs, and blocking the CD47-SIRPα pathway (141). Nanomedicines, natural drug components, and synthetic drugs can each reprogram TAMs through the cGAS-STING, IL-6R/JAK, HDGF/IL-4/JAK1/STAT3, and STAT6 signaling pathways.

Due to the impact of TAMs on tumor progression, drug development targeting TAMs has gained a lot of attention. To date, the therapeutic strategies targeting TAMs in the TME are primarily divided into four types (**Figure 4**). Drug development targeting macrophage phenotypes regulation is a promising direction. In terms of synthetic some studies have found that the TKIs imatinib and gefitinib can prevent macrophage M2 polarization by inhibiting STAT6 phosphorylation (142, 143). Polarized M2 macrophages can respond to external stimuli such as cytokines to repolarize into an M1 phenotype. Wei and colleagues found that thymosin alpha1 can reprogram TAMs towards an M1 phenotype through interaction with TLR7 and TLR8 on the lysosomal membrane (144). Additionally, more natural biological compounds have been discovered to be associated with macrophage polarization (**Table 1**). With the development of biotechnology, comprehensive analyses of the temporal changes in cell metabolism, function, and signaling pathways during the induction stages of M1 and M2 polarization have been facilitated. Integrating multidimensional datasets, new mechanisms of macrophage polarization have been proposed (154). This also provides more target options for drug development.

**Table 1:** Examples of natural compounds that alter the phenotype of TAMs in LC.

Natural drug compound	Signal pathway	Macrophage polarization	Ref.
Dioscin	JNK and STAT3	M1↑	(145)
Lapachol	NF-κB	M2↓	(146)
MTE	HDGF/IL-4/JAK1/STAT3	M2↓	(147)
VA	IL-6R/JAK	M1↑	(148)
DHA	AKT/mTOR	M1↑ M2↓	(149)
MEMA	STAT6 and STAT3	M2↓	(150)
CT	TLR7/MyD88/NF-κB	M1↑	(151)
Sang	WNT/β-Catenin	M2↓	(152)
AS-IV	AMPK	M2↓	(153)

Ref., references; MTE, marsdenia tenacissima extract; VA, vanillic acid; DHA, dihydroartemisinin; MEMA, methylene chloride extract of Morus alba L.; CT, cryptotanshinone; Sang, sanguinarine; AS-IV, astragaloside IV. The specific meanings of the symbols ↑,↓ are as follows: ↓: Decrease; ↑: Increase.

In summary, developing drugs to regulate macrophages for anti-tumor phenotypes is a crucial work with significant clinical application prospects. However, substantial efforts are still needed for drug target screening and further basic research. Advancements in biotechnology are advantageous for promoting drug synthesis and target screening.

## 7 Progress in NDDSs targeting TAMs in LC

At present, nanotechnology is a focal point in the field of drug delivery systems, particularly prominent in targeted therapies. Nanoparticles (NPs) possess modifiable features such as shape, size, and functional properties (155). In the characteristics of NPs, the mechanism of interaction with macrophages has been extensively studied in terms of density. In summary, the high density of NPs engulfed by macrophages leads to reduced mobility of the cells themselves (156). NPs can collaborate with drugs, indicating that NDDSs have the potential to propel drug therapy into the era of precision medicine (157). In various types of diseases, there is substantial evidence that NDDSs improve treatment outcomes by modulating the phenotype of immune cells (158–160). NDDSs induce the repolarization of anti-inflammatory M2 macrophages to pro-inflammatory M1 phenotype, which enhances the anti-tumor effect (161, 162). NDDSs based on various nanomaterials have greatly transformed the field of TAM-related immunotherapy (53, 163).

A novel type of silver nano-cluster (AgNCs) can initiate M1-like polarization of macrophages through the toll-like receptor 4 (TLR-4) signaling axis (164). Nano-Doxorubicin (Nano-DOX) is a drug that has been shown to stimulate the immunogenicity of tumor cells, effectively reactivating TAMs into an anti-tumor M1 phenotype (165). Another type of NPs extracted from cuttlefish ink (CINPs), which can effectively reprogram TAMs from an immunosuppressive M2-like phenotype to an anti-tumor M1-like phenotype (166). Furthermore, plant-derived mitochondrial DNA (mtDNA), encapsulated in nanovesicles derived from artemisinin (ADNV), when internalized into TAMs, induces the major effector molecules of the cGAS-STING pathway, driving the M1-like polarization (167).

Furthermore, due to the direct communication of the lung with the external environment, inhalable NDDSs have also garnered significant attention. One inhalable nanoscale formulation, DFHC (Dex@Fe-HMSNs@Ct), interacts with the CD206 receptor highly expressed in M2-like macrophages, facilitating its uptake by TAMs. Subsequently, through endogenous iron metabolism, DFHC triggers iron death in cancer stem cells (CSCs) (168). The liposome-based NPs (AeroNP-CDN) effectively alleviate the immunosuppressive TME by reprogramming TAMs from the M2 to M1 phenotype (169).

Macrophage-based NDDSs represent an emerging cancer treatment strategy. Also, it has the potential to overcome various clinical challenges and pave the way for a new era of drug therapy. Unfortunately, there are still some barriers and challenges that limit its clinical application. For example, tracking and effectively delivering drugs to the TME remains difficult, necessitating a deeper understanding of the dynamic changes in the microenvironment and the exploration of novel nanomaterials. Furthermore, due to the plasticity of macrophages and the discovery of more subtypes, controlling their states also presents various challenges.

## 8 Prospects and outlooks

Macrophages are an important component of the TME in LC, and their infiltration levels depend on the severity and staging of the disease. Once inside the TME, macrophages are exposed to a variety of signals and stimuli that shape their phenotype and function. Depending on the dynamic balance of these signals, macrophages can exist in different activation states ranging from M1-like to M2-like. Generally, M1 macrophages have anti-tumor functions, while M2 macrophages promote tumor functions. Additionally, the signaling molecules associated with TAM polarization can serve as potential prognostic biomarkers for LC. Blocking or enhancing signaling pathways that polarize TAMs can enhance the body’s anti-tumor capabilities and have a synergistic effect with drug therapy. Due to the unique heterogeneity of TAMs, the timing and process of macrophage recruitment and polarization are not yet clear. The complexity and diversity of macrophage polarization make it challenging to find drugs that are effective against specific types of tumors. Therefore, further research is needed to effectively design drugs targeted at macrophages. Simultaneously, the upstream and downstream pathways can also serve as therapeutic targets to regulate the function of macrophages. Particularly, utilizing genetic engineering to genetically reprogram macrophages, transforming anti-tumor phenotypes. In both *in vitro* and *in vivo* models, an increasing number of natural plant ingredients have been confirmed to regulate macrophage phenotype. Due to the advantages of diverse types and low toxicity of natural plant ingredients, they have attracted attention. However, some metabolic mechanisms remain unclear, requiring further in-depth basic research. Promoting the polarization of TAMs towards an anti-tumor phenotype represents a very promising research direction. Epigenetics plays a crucial role in controlling the phenotypes of macrophages in TEM. Therefore, drugs that target epigenetic signaling pathways show promising potential for cancer treatment. Precision medicine is essential for advancing epigenetic therapies in the future. With hundreds of potential targets, epigenetic changes are prevalent abnormalities in cancer, occurring either early as foundational mutations or later to drive distinct subpopulations. Understanding how these alterations interact can aid in identifying mutations suitable for targeted therapies. The similarity of normal cell receptors can lead to the failure of targeting immune-suppressive macrophages, resulting in side effects and toxicity. It is essential to further decipher the functional characteristics of TAMs and the signaling crosstalk with various types of cells, which aids in identifying specific targets. Due to advancements in artificial intelligence and molecular biology, more specific functional subtypes of TAMs have been defined. Drugs designed specifically for these functionally distinct macrophage subtypes may be more advantageous for the treatment of LC.

Nanomedicine is a promising therapeutic approach and is continually being improved and developed. Nanomedicine strategies often exploit the increased expression of receptors on TAMs, which can change as the tumor progresses. Though NDDSs have shown some effectiveness in cancer immunotherapy and drug delivery, numerous challenges still need addressing. Critical factors such as the uptake of NPs by macrophages and the metabolic processes are essential mechanisms that require further clarification. Due to the characteristics of catalytic activity and modifiability of NPs, they bring unlimited possibilities for solving complex clinical problems. Given the complexity of the TME, cancer immunotherapies that solely focus on targeting TAM receptors are not enough to completely eradicate tumors. Chimeric antigen receptor (CAR) is a modified receptor protein that endows immune cells with the ability to recognize specific antigens on the surface of cancer cells, thereby stimulating immune cells to eliminate cancer cells (170). Macrophages can activate T-cells by presenting antigens, activating the adaptive immune response, and enhancing the anti-tumor effect. In terms of safety, macrophages have a limited time in circulation, and the risk of graft-versus-host disease (GvHD) is relatively low. Therefore, macrophages have the potential to be developed into chimeric antigen receptor macrophage (CAR-M) therapy, serving as a form of anti-tumor immune cell therapy (171). CAR-M has been demonstrated to have promising anti-tumor effects *in vitro* cell and animal experiments (172, 173). In comparison to CAR-T, CAR-M offers several advantages. CAR-M can reduce the proportion of TAMs, alter TAMs’ cellular phenotypes, and positively impact tumor treatment (172). In addition to its role in engulfing tumor cells, CAR-M also enhances antigen presentation capabilities and promotes T-cell cytotoxicity (174). The circulation time of CAR-M is limited, resulting in lower side effects on other organs (172). Recently, Shen and his colleagues developed an efficient monolayer culture system capable of producing approximately 6000 macrophages from a single human pluripotent stem cell (hPSC) within a 3-week period. Utilizing a CAR structure screening approach, the research team *in vitro* generated macrophages with stable CAR expression and potent tumor-killing activity (175). Zhang et al. designed antigen-dependent polarization of second-generation iPSC-derived CAR-M, elucidating their antigen-dependent polarization and activation mechanisms, as well as their tumor-killing mechanism through “phagoptosis” (176). These findings provide a more solid theoretical foundation for the application of CAR-M in immunotherapy for solid tumors, supporting its translation into clinical applications. Integrating these therapies with other immunotherapeutic approaches, like PD-L1 or CAR therapies, presents an effective strategy to tackle this problem. Epigenetic factors can be used to express antigens, gene editing, or regulate disease-related proteins. Among them, mRNA nanomedicine can directly produce the required proteins in the body, avoiding the complex process of drug development and production. NDDSs introduce innovative approaches for precise targeting and combination therapies. Developing combination therapies that leverage the properties of NPs and the TME to achieve synergistic effects and minimize adverse reactions is a pressing challenge. To overcome these obstacles, it’s crucial to design NPs and drugs with even greater targeting capabilities, further transforming macrophages into powerful tools against solid tumors. At the same time, conducting interdisciplinary basic experiments is necessary, as it will be more conducive to understanding various mechanisms.

## 9 Conclusion

Specific stimuli within the TME can induce macrophages activation and differentiation into various functional subgroups. This process is associated with extensive epigenetic modifications, transcriptional reprogramming, and metabolic changes. Macrophages are a prevalent component of the TME and engage in complex interactions with cancer cells, the matrix, and immune active cells. Modulating the macrophage phenotype is an important direction in the development of anti-tumor drugs targeting TAMs. Macrophage polarization is a complex process of multifactorial interactions, regulated by various intracellular signaling molecules and their pathways. Drugs are categorized based on their effects on macrophage phenotypes, mainly including inhibitors of M2-like macrophage polarization, inducers of M2-like to M1-like macrophage transformation, and promoters of M1-like macrophage polarization. Significant progress has been made in drug development targeting macrophages. Natural plant ingredients as immunomodulatory drugs targeting macrophages have promising prospects for application. However, numerous obstacles remain, such as exploring specific target drugs in complex microenvironments, mitigating the adverse effects on other immune cells. Moreover, the adverse reactions and mechanisms in macrophage-targeted therapy and the rational combination of drugs require further research. The catalytic and modifiability of NPs often provide solutions to various clinical problems. NDDSs have created conditions for the precise delivery of drugs, but a deeper investigation is needed for drugs to better match with NPs. In summary, macrophages present a promising therapeutic target, especially by enhancing the anti-tumor effect through altering their phenotype. The complexity of the TME presents obstacles for drug development, with more mechanistic studies awaiting completion.

## Author contributions

WL: Investigation, Project administration, Visualization, Writing – original draft. QY: Investigation, Project administration, Visualization, Writing – original draft. ML: Writing – original draft. XH: Writing – original draft. CS: Writing – review & editing. YRL: Writing – review & editing. YT: Writing – review & editing. YLi: Conceptualization, Formal analysis, Funding acquisition, Writing – review & editing. ZD: Conceptualization, Funding acquisition, Supervision, Validation, Writing – review & editing. YLu: Conceptualization, Investigation, Supervision, Validation, Visualization, Writing – review & editing.
